# 5-Bromo-3-cyclo­hexyl­sulfinyl-2-methyl-1-benzofuran

**DOI:** 10.1107/S1600536811003242

**Published:** 2011-01-29

**Authors:** Hong Dae Choi, Pil Ja Seo, Byeng Wha Son, Uk Lee

**Affiliations:** a Department of Chemistry, Dongeui University, San 24 Kaya-dong Busanjin-gu, Busan 614-714, Republic of Korea; b Department of Chemistry, Pukyong National University, 599-1 Daeyeon 3-dong, Nam-gu, Busan 608-737, Republic of Korea

## Abstract

In the asymmetric unit of the title compound, C_15_H_17_BrO_3_S, there are two independent mol­ecules. The cyclo­hexane rings in each adopt classic chair conformations. In the crystal, mol­ecules are linked by weak inter­molecular C—H⋯O hydrogen bonds and aromatic π–π inter­actions between the furan rings of symmetry-related mol­ecules [centroid–centroid distance = 3.555 (2) Å].

## Related literature

For the pharmacological activity of benzofuran compounds, see: Aslam *et al.* (2006 ▶); Galal *et al.* (2009 ▶); Khan *et al.* (2005 ▶). For natural products with benzofuran rings, see: Akgul & Anil (2003 ▶); Soekamto *et al.* (2003 ▶). For related structures, see: Choi *et al.* (2007 ▶); Seo *et al.* (2009 ▶).
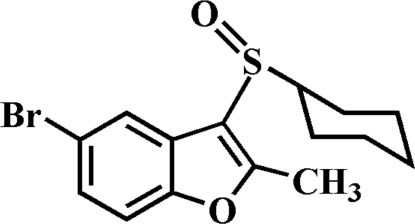


## Experimental

### 

#### Crystal data

C_15_H_17_BrO_2_S
*M* *_r_* = 341.26
Monoclinic, 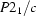

*a* = 12.1842 (2) Å
*b* = 9.0281 (1) Å
*c* = 26.6191 (4) Å
β = 97.702 (1)°
*V* = 2901.69 (7) Å^3^
*Z* = 8
Mo *K*α radiation
μ = 2.97 mm^−1^
*T* = 173 K
0.31 × 0.22 × 0.18 mm

#### Data collection

Bruker SMART APEXII CCD diffractometer
Absorption correction: multi-scan (*SADABS*; Bruker, 2009 ▶) *T*_min_ = 0.579, *T*_max_ = 0.746
26831 measured reflections
6674 independent reflections
4944 reflections with *I* > 2σ(*I*)
*R*_int_ = 0.041

#### Refinement

*R*[*F* ^2^ > 2σ(*F* ^2^)] = 0.035
*wR*(*F* ^2^) = 0.088
*S* = 1.04
6674 reflections
345 parameters
H-atom parameters constrained
Δρ_max_ = 0.41 e Å^−3^
Δρ_min_ = −0.56 e Å^−3^

### 

Data collection: *APEX2* (Bruker, 2009 ▶); cell refinement: *SAINT* (Bruker, 2009 ▶); data reduction: *SAINT*; program(s) used to solve structure: *SHELXS97* (Sheldrick, 2008 ▶); program(s) used to refine structure: *SHELXL97* (Sheldrick, 2008 ▶); molecular graphics: *ORTEP-3* (Farrugia, 1997 ▶) and *DIAMOND* (Brandenburg, 1998 ▶); software used to prepare material for publication: *SHELXL97*.

## Supplementary Material

Crystal structure: contains datablocks global, I. DOI: 10.1107/S1600536811003242/lh5201sup1.cif

Structure factors: contains datablocks I. DOI: 10.1107/S1600536811003242/lh5201Isup2.hkl

Additional supplementary materials:  crystallographic information; 3D view; checkCIF report

## Figures and Tables

**Table 1:** Hydrogen-bond geometry (Å, °)

*D*—H⋯*A*	*D*—H	H⋯*A*	*D*⋯*A*	*D*—H⋯*A*
C5—H5⋯O2^i^	0.95	2.56	3.495 (3)	170
C25—H25⋯O2	1.00	2.57	3.409 (3)	142

Symmetry code: (i) 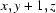.

**Fig. 1.**
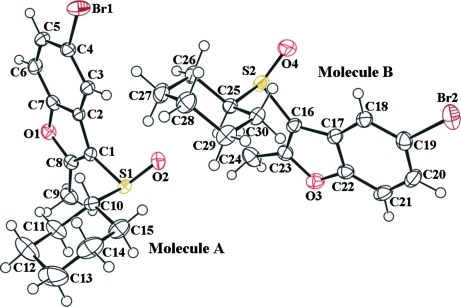
The molecular structure of the title compound with displacement ellipsoids drawn at the 50% probability level. H atoms are presented as a small spheres of arbitrary radius.

**Fig. 2.**
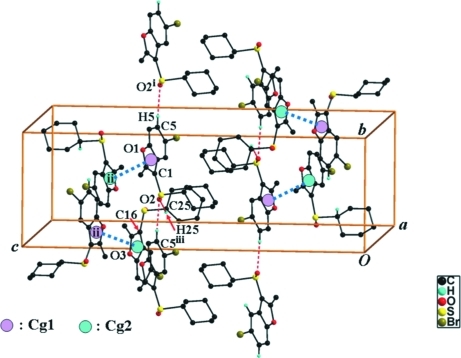
A view of the C—H···O and π–π interactions (dotted lines) in the crystal packing of the title compound. [Symmetry codes: (i) x, y + 1, z; (ii) - x + 1, y + 1/2, - z + 3/2; (iii) x, - 1 +y,z .]

## supplementary crystallographic information

### Comment

Many compounds involving a benzofuran ring have potential pharmacological properties such as antifungal, antitumor and antiviral, and antimicrobial activities (Aslam *et al.*, 2006; Galal *et al.*, 2009, Khan *et al.*, 2005). These compounds occur in a wide range of natural products (Akgul & Anil, 2003; Soekamto *et al.*, 2003). As a part of our ongoing study of the substituent effect on the solid state structures of 5-bromo-2-methyl-3-methylsufinyl-1-benzofuran analogues (Choi *et al.*, 2007, Seo *et al.*, 2009), we report herein the crystal structure of the title compound.

The asymmetric unit of the title compound is shown in Fig. 1. There are two independent molecules [A and B] in which the benzofuran unit is essentially planar in each with a mean deviation of 0.013 (2) Å and 0.014 (2) Å for A and B, respectively from the least-squares plane defined by the nine constituent atoms. The cyclohexyl rings are in the chair form. The molecular packing (Fig. 2) is stabilized by weak intermolecular C—H···O hydrogen bonds; the first one between a benzene H atom and the oxygen atom of the S═O unit (Table 1; C5—H5···O2^i^), and the second one between a cyclohexyl H atom and the oxygen atom of the S═O unit (Table 1; C25—H25···O2). An intramolecular hydrogen bond exists between a cyclohexyl H atom and the oxygen atom of the S═O unit (Table 1; C25—H25···O2). Further stabilization is provided by aromatic π–π interactions between the furan rings of symmetry related molecules, with a Cg1···Cg2(1-x,1/2+y,3/2-z) distance of 3.555 (2) Å (Cg1 and Cg2 are the centroids of the C1/C2/C7/O1/C8 and C16/C17/C22/O3/C23 furan rings, respectively).

### Experimental

77% 3-chloroperoxybenzoic acid (224 mg, 1.0 mmol) was added in small portions to a stirred solution of 5-bromo-3-cyclohexylsulfanyl-2-methyl-1-benzofuran (293 mg, 0.9 mmol) in dichloromethane (40 mL) at 273 K. After being stirred at room temperature for 3h, the mixture was washed with saturated sodium bicarbonate solution and the organic layer was separated, dried over magnesium sulfate, filtered and concentrated at reduced pressure. The residue was purified by column chromatography (hexane–ethyl acetate, 2:1 v/v) to afford the title compound as a colorless solid [yield 72%, m.p. 388–389 K; *R*_f_ = 0.45 (hexane–ethyl acetate, 2:1 v/v)]. Single crystals suitable for X-ray diffraction were prepared by slow evaporation of a solution of the title compound in ethyl acetate at room temperature.

### Refinement

All H atoms were positioned geometrically and refined using a riding model, with C—H = 0.95 Å   for aryl, 1.00 Å for methine, 0.99 Å for methylene, and 0.98 Å for methyl H atoms, respectively. *U*_iso_(H) =1.2*U*_eq_(C) for aryl,  methine and methylene, and 1.5*U*_eq_(C) for methyl H atoms.

### Crystal data

**Table:**

C_15_H_17_BrO_2_S	*F*(000) = 1392
*M**_r_* = 341.26	*D*_x_ = 1.562 Mg m^−^^3^
Monoclinic, *P*2_1_/*c*	Mo *K*α radiation, λ = 0.71073 Å
Hall symbol: -P 2ybc	Cell parameters from 7002 reflections
*a* = 12.1842 (2) Å	θ = 2.4–24.9°
*b* = 9.0281 (1) Å	µ = 2.97 mm^−^^1^
*c* = 26.6191 (4) Å	*T* = 173 K
β = 97.702 (1)°	Block, colourless
*V* = 2901.69 (7) Å^3^	0.31 × 0.22 × 0.18 mm
*Z* = 8	

### Data collection

**Table:**

Bruker SMART APEXII CCD diffractometer	6674 independent reflections
Radiation source: rotating anode	4944 reflections with *I* > 2σ(*I*)
graphite multilayer	*R*_int_ = 0.041
Detector resolution: 10.0 pixels mm^-1^	θ_max_ = 27.5°, θ_min_ = 1.5°
φ and ω scans	*h* = −15→15
Absorption correction: multi-scan (*SADABS*; Bruker, 2009)	*k* = −10→11
*T*_min_ = 0.579, *T*_max_ = 0.746	*l* = −32→34
26831 measured reflections	

### Refinement

**Table:**

Refinement on *F*^2^	Primary atom site location: structure-invariant direct methods
Least-squares matrix: full	Secondary atom site location: difference Fourier map
*R*[*F*^2^ > 2σ(*F*^2^)] = 0.035	Hydrogen site location: difference Fourier map
*wR*(*F*^2^) = 0.088	H-atom parameters constrained
*S* = 1.04	*w* = 1/[σ^2^(*F*_o_^2^) + (0.0465*P*)^2^ + 0.1726*P*] where *P* = (*F*_o_^2^ + 2*F*_c_^2^)/3
6674 reflections	(Δ/σ)_max_ = 0.001
345 parameters	Δρ_max_ = 0.41 e Å^−^^3^
0 restraints	Δρ_min_ = −0.56 e Å^−^^3^

### Special details

**Table:**

Geometry. All esds (except the esd in the dihedral angle between two l.s. planes) are estimated using the full covariance matrix. The cell esds are taken into account individually in the estimation of esds in distances, angles and torsion angles; correlations between esds in cell parameters are only used when they are defined by crystal symmetry. An approximate (isotropic) treatment of cell esds is used for estimating esds involving l.s. planes.
Refinement. Refinement of F^2^ against ALL reflections. The weighted R-factor wR and goodness of fit S are based on F^2^, conventional R-factors R are based on F, with F set to zero for negative F^2^. The threshold expression of F^2^ > 2sigma(F^2^) is used only for calculating R-factors(gt) etc. and is not relevant to the choice of reflections for refinement. R-factors based on F^2^ are statistically about twice as large as those based on F, and R- factors based on ALL data will be even larger.

### Fractional atomic coordinates and isotropic or equivalent isotropic displacement parameters (Å^2^)

**Table:**

	*x*	*y*	*z*	*U*_iso_*/*U*_eq_	
Br1	0.46756 (2)	0.86266 (3)	0.592298 (11)	0.04012 (9)	
Br2	−0.07658 (2)	−0.22211 (3)	0.590592 (11)	0.04406 (10)	
S1	0.73629 (5)	0.29635 (6)	0.65910 (2)	0.02434 (14)	
S2	0.27538 (5)	0.26776 (7)	0.66785 (2)	0.02864 (15)	
O1	0.87123 (13)	0.65573 (18)	0.72524 (6)	0.0277 (4)	
O2	0.61309 (14)	0.28891 (19)	0.65360 (7)	0.0350 (4)	
O3	0.36105 (14)	−0.13307 (18)	0.71689 (6)	0.0313 (4)	
O4	0.15602 (15)	0.3032 (2)	0.65511 (7)	0.0401 (5)	
C1	0.77497 (18)	0.4739 (2)	0.68179 (8)	0.0211 (5)	
C2	0.71675 (18)	0.6108 (2)	0.66932 (8)	0.0202 (5)	
C3	0.62204 (19)	0.6512 (3)	0.63733 (8)	0.0230 (5)	
H3	0.5777	0.5803	0.6175	0.028*	
C4	0.59557 (19)	0.8007 (3)	0.63584 (9)	0.0250 (5)	
C5	0.6577 (2)	0.9072 (3)	0.66440 (9)	0.0299 (6)	
H5	0.6357	1.0082	0.6619	0.036*	
C6	0.7517 (2)	0.8665 (3)	0.69656 (9)	0.0294 (6)	
H6	0.7951	0.9371	0.7169	0.035*	
C7	0.77938 (19)	0.7179 (3)	0.69753 (9)	0.0239 (5)	
C8	0.86624 (19)	0.5065 (3)	0.71497 (8)	0.0249 (5)	
C9	0.9604 (2)	0.4162 (3)	0.73881 (10)	0.0353 (6)	
H9A	0.9366	0.3131	0.7413	0.053*	
H9B	0.9860	0.4546	0.7728	0.053*	
H9C	1.0210	0.4210	0.7181	0.053*	
C10	0.77442 (19)	0.3168 (3)	0.59584 (9)	0.0234 (5)	
H10	0.7406	0.4100	0.5805	0.028*	
C11	0.8996 (2)	0.3278 (3)	0.59887 (10)	0.0394 (7)	
H11A	0.9343	0.2411	0.6174	0.047*	
H11B	0.9257	0.4182	0.6179	0.047*	
C12	0.9348 (3)	0.3334 (4)	0.54597 (12)	0.0561 (9)	
H12A	0.9079	0.4266	0.5290	0.067*	
H12B	1.0166	0.3327	0.5489	0.067*	
C13	0.8886 (3)	0.2023 (4)	0.51411 (12)	0.0612 (10)	
H13A	0.9092	0.2113	0.4795	0.073*	
H13B	0.9211	0.1094	0.5293	0.073*	
C14	0.7638 (3)	0.1964 (3)	0.51127 (10)	0.0487 (8)	
H14A	0.7354	0.1095	0.4909	0.058*	
H14B	0.7313	0.2866	0.4941	0.058*	
C15	0.7284 (2)	0.1857 (3)	0.56366 (10)	0.0380 (6)	
H15A	0.7561	0.0920	0.5801	0.046*	
H15B	0.6465	0.1853	0.5608	0.046*	
C16	0.28708 (19)	0.0766 (3)	0.68148 (8)	0.0242 (5)	
C17	0.2120 (2)	−0.0437 (3)	0.66480 (9)	0.0245 (5)	
C18	0.11013 (19)	−0.0572 (3)	0.63480 (8)	0.0255 (5)	
H18	0.0730	0.0264	0.6189	0.031*	
C19	0.0653 (2)	−0.1977 (3)	0.62917 (9)	0.0294 (6)	
C20	0.1191 (2)	−0.3231 (3)	0.65164 (10)	0.0344 (6)	
H20	0.0860	−0.4180	0.6461	0.041*	
C21	0.2188 (2)	−0.3094 (3)	0.68142 (10)	0.0343 (6)	
H21	0.2562	−0.3930	0.6972	0.041*	
C22	0.2629 (2)	−0.1690 (3)	0.68765 (9)	0.0264 (5)	
C23	0.3726 (2)	0.0180 (3)	0.71287 (9)	0.0280 (5)	
C24	0.4728 (2)	0.0833 (3)	0.74241 (10)	0.0409 (7)	
H24A	0.5345	0.0792	0.7223	0.061*	
H24B	0.4921	0.0272	0.7739	0.061*	
H24C	0.4581	0.1867	0.7505	0.061*	
C25	0.33763 (19)	0.2705 (2)	0.60925 (8)	0.0228 (5)	
H25	0.4133	0.2260	0.6165	0.027*	
C26	0.3509 (2)	0.4326 (3)	0.59471 (9)	0.0308 (6)	
H26A	0.2773	0.4808	0.5888	0.037*	
H26B	0.3963	0.4853	0.6228	0.037*	
C27	0.4067 (2)	0.4425 (3)	0.54673 (9)	0.0332 (6)	
H27A	0.4125	0.5476	0.5369	0.040*	
H27B	0.4825	0.4015	0.5536	0.040*	
C28	0.3413 (2)	0.3575 (3)	0.50351 (10)	0.0354 (6)	
H28A	0.3803	0.3627	0.4732	0.043*	
H28B	0.2675	0.4035	0.4948	0.043*	
C29	0.3274 (2)	0.1960 (3)	0.51799 (9)	0.0320 (6)	
H29A	0.4009	0.1474	0.5233	0.038*	
H29B	0.2813	0.1442	0.4899	0.038*	
C30	0.2729 (2)	0.1827 (3)	0.56638 (9)	0.0268 (5)	
H30A	0.1960	0.2204	0.5599	0.032*	
H30B	0.2700	0.0772	0.5763	0.032*	

### Atomic displacement parameters (Å^2^)

**Table:**

	*U*^11^	*U*^22^	*U*^33^	*U*^12^	*U*^13^	*U*^23^
Br1	0.03958 (16)	0.03535 (16)	0.04353 (17)	0.01601 (13)	−0.00135 (12)	0.00518 (12)
Br2	0.03332 (16)	0.0532 (2)	0.04619 (18)	−0.01426 (14)	0.00714 (13)	−0.00397 (14)
S1	0.0269 (3)	0.0171 (3)	0.0293 (3)	−0.0007 (2)	0.0049 (2)	0.0014 (2)
S2	0.0371 (4)	0.0203 (3)	0.0308 (3)	−0.0019 (3)	0.0129 (3)	−0.0023 (3)
O1	0.0235 (9)	0.0308 (9)	0.0279 (9)	−0.0036 (7)	0.0006 (7)	−0.0047 (7)
O2	0.0271 (9)	0.0309 (10)	0.0486 (11)	−0.0076 (8)	0.0112 (8)	−0.0029 (8)
O3	0.0353 (10)	0.0293 (10)	0.0293 (9)	0.0065 (8)	0.0042 (8)	0.0044 (7)
O4	0.0382 (11)	0.0323 (10)	0.0534 (12)	0.0061 (9)	0.0198 (9)	0.0080 (9)
C1	0.0218 (11)	0.0199 (12)	0.0220 (12)	−0.0012 (9)	0.0039 (9)	0.0018 (9)
C2	0.0228 (11)	0.0183 (11)	0.0208 (11)	0.0000 (9)	0.0082 (9)	0.0006 (9)
C3	0.0247 (12)	0.0204 (12)	0.0241 (12)	0.0001 (10)	0.0042 (10)	−0.0001 (10)
C4	0.0259 (13)	0.0246 (13)	0.0254 (12)	0.0037 (10)	0.0072 (10)	0.0033 (10)
C5	0.0348 (14)	0.0185 (12)	0.0387 (15)	0.0010 (11)	0.0134 (12)	−0.0005 (11)
C6	0.0327 (14)	0.0230 (13)	0.0335 (14)	−0.0071 (11)	0.0084 (11)	−0.0091 (11)
C7	0.0210 (12)	0.0294 (13)	0.0216 (12)	−0.0024 (10)	0.0042 (9)	−0.0036 (10)
C8	0.0259 (12)	0.0292 (13)	0.0199 (12)	0.0012 (10)	0.0044 (9)	0.0018 (10)
C9	0.0253 (13)	0.0486 (16)	0.0312 (14)	0.0070 (12)	0.0008 (11)	0.0059 (12)
C10	0.0250 (12)	0.0206 (12)	0.0248 (12)	0.0030 (10)	0.0039 (10)	−0.0004 (9)
C11	0.0257 (14)	0.0558 (18)	0.0375 (15)	0.0042 (13)	0.0071 (12)	−0.0041 (13)
C12	0.0397 (17)	0.086 (2)	0.0462 (19)	0.0061 (18)	0.0204 (14)	0.0000 (18)
C13	0.085 (3)	0.065 (2)	0.0386 (18)	0.024 (2)	0.0271 (18)	−0.0063 (16)
C14	0.076 (2)	0.0350 (16)	0.0340 (16)	0.0026 (16)	0.0017 (15)	−0.0115 (13)
C15	0.0502 (17)	0.0280 (14)	0.0344 (15)	−0.0021 (13)	0.0009 (13)	−0.0073 (12)
C16	0.0299 (13)	0.0203 (12)	0.0240 (12)	−0.0002 (10)	0.0091 (10)	−0.0014 (10)
C17	0.0309 (13)	0.0211 (12)	0.0235 (12)	0.0006 (10)	0.0110 (10)	0.0026 (10)
C18	0.0289 (13)	0.0240 (13)	0.0247 (13)	0.0003 (10)	0.0074 (10)	0.0036 (10)
C19	0.0293 (13)	0.0330 (14)	0.0273 (13)	−0.0056 (11)	0.0089 (10)	−0.0039 (11)
C20	0.0456 (17)	0.0212 (13)	0.0396 (16)	−0.0063 (12)	0.0168 (13)	−0.0025 (11)
C21	0.0450 (16)	0.0210 (13)	0.0397 (16)	0.0053 (12)	0.0163 (13)	0.0062 (11)
C22	0.0292 (13)	0.0255 (13)	0.0255 (13)	0.0035 (11)	0.0077 (10)	0.0014 (10)
C23	0.0300 (13)	0.0291 (13)	0.0264 (13)	−0.0010 (11)	0.0097 (10)	−0.0024 (11)
C24	0.0311 (14)	0.0576 (18)	0.0338 (15)	−0.0026 (14)	0.0037 (12)	−0.0087 (14)
C25	0.0241 (12)	0.0208 (12)	0.0243 (12)	−0.0005 (10)	0.0056 (10)	0.0000 (10)
C26	0.0340 (14)	0.0230 (13)	0.0373 (15)	−0.0050 (11)	0.0115 (11)	−0.0014 (11)
C27	0.0388 (15)	0.0271 (14)	0.0354 (15)	−0.0052 (12)	0.0107 (12)	0.0047 (11)
C28	0.0452 (16)	0.0323 (15)	0.0292 (14)	−0.0009 (13)	0.0065 (12)	0.0069 (11)
C29	0.0430 (16)	0.0286 (14)	0.0239 (13)	−0.0014 (12)	0.0028 (11)	−0.0005 (11)
C30	0.0303 (13)	0.0208 (12)	0.0290 (13)	−0.0037 (10)	0.0027 (10)	−0.0011 (10)

### Geometric parameters (Å, °)

**Table:**

Br1—C4	1.899 (2)	C13—H13B	0.9900
Br2—C19	1.902 (3)	C14—C15	1.517 (4)
S1—O2	1.4901 (18)	C14—H14A	0.9900
S1—C1	1.755 (2)	C14—H14B	0.9900
S1—C10	1.815 (2)	C15—H15A	0.9900
S2—O4	1.483 (2)	C15—H15B	0.9900
S2—C16	1.765 (2)	C16—C23	1.353 (3)
S2—C25	1.824 (2)	C16—C17	1.451 (3)
O1—C8	1.374 (3)	C17—C18	1.388 (3)
O1—C7	1.375 (3)	C17—C22	1.390 (3)
O3—C22	1.376 (3)	C18—C19	1.382 (3)
O3—C23	1.377 (3)	C18—H18	0.9500
C1—C8	1.357 (3)	C19—C20	1.401 (4)
C1—C2	1.442 (3)	C20—C21	1.364 (4)
C2—C3	1.388 (3)	C20—H20	0.9500
C2—C7	1.388 (3)	C21—C22	1.378 (3)
C3—C4	1.387 (3)	C21—H21	0.9500
C3—H3	0.9500	C23—C24	1.483 (3)
C4—C5	1.386 (3)	C24—H24A	0.9800
C5—C6	1.385 (4)	C24—H24B	0.9800
C5—H5	0.9500	C24—H24C	0.9800
C6—C7	1.383 (3)	C25—C30	1.521 (3)
C6—H6	0.9500	C25—C26	1.528 (3)
C8—C9	1.480 (3)	C25—H25	1.0000
C9—H9A	0.9800	C26—C27	1.528 (3)
C9—H9B	0.9800	C26—H26A	0.9900
C9—H9C	0.9800	C26—H26B	0.9900
C10—C11	1.520 (3)	C27—C28	1.517 (4)
C10—C15	1.524 (3)	C27—H27A	0.9900
C10—H10	1.0000	C27—H27B	0.9900
C11—C12	1.527 (4)	C28—C29	1.523 (3)
C11—H11A	0.9900	C28—H28A	0.9900
C11—H11B	0.9900	C28—H28B	0.9900
C12—C13	1.519 (5)	C29—C30	1.532 (3)
C12—H12A	0.9900	C29—H29A	0.9900
C12—H12B	0.9900	C29—H29B	0.9900
C13—C14	1.513 (5)	C30—H30A	0.9900
C13—H13A	0.9900	C30—H30B	0.9900
			
O2—S1—C1	107.19 (10)	C10—C15—H15A	109.7
O2—S1—C10	107.04 (11)	C14—C15—H15B	109.7
C1—S1—C10	97.81 (11)	C10—C15—H15B	109.7
O4—S2—C16	107.92 (11)	H15A—C15—H15B	108.2
O4—S2—C25	107.99 (11)	C23—C16—C17	107.3 (2)
C16—S2—C25	99.08 (10)	C23—C16—S2	122.95 (19)
C8—O1—C7	106.39 (17)	C17—C16—S2	129.71 (18)
C22—O3—C23	106.25 (18)	C18—C17—C22	119.5 (2)
C8—C1—C2	107.4 (2)	C18—C17—C16	136.0 (2)
C8—C1—S1	125.56 (18)	C22—C17—C16	104.4 (2)
C2—C1—S1	127.08 (17)	C19—C18—C17	116.9 (2)
C3—C2—C7	120.1 (2)	C19—C18—H18	121.6
C3—C2—C1	135.2 (2)	C17—C18—H18	121.6
C7—C2—C1	104.76 (19)	C18—C19—C20	122.7 (2)
C4—C3—C2	116.4 (2)	C18—C19—Br2	118.84 (19)
C4—C3—H3	121.8	C20—C19—Br2	118.43 (19)
C2—C3—H3	121.8	C21—C20—C19	120.2 (2)
C5—C4—C3	123.5 (2)	C21—C20—H20	119.9
C5—C4—Br1	118.29 (18)	C19—C20—H20	119.9
C3—C4—Br1	118.24 (18)	C20—C21—C22	117.1 (2)
C6—C5—C4	120.1 (2)	C20—C21—H21	121.4
C6—C5—H5	120.0	C22—C21—H21	121.4
C4—C5—H5	120.0	O3—C22—C21	125.5 (2)
C7—C6—C5	116.6 (2)	O3—C22—C17	111.0 (2)
C7—C6—H6	121.7	C21—C22—C17	123.5 (2)
C5—C6—H6	121.7	C16—C23—O3	111.0 (2)
O1—C7—C6	125.8 (2)	C16—C23—C24	133.1 (2)
O1—C7—C2	110.8 (2)	O3—C23—C24	116.0 (2)
C6—C7—C2	123.4 (2)	C23—C24—H24A	109.5
C1—C8—O1	110.7 (2)	C23—C24—H24B	109.5
C1—C8—C9	132.9 (2)	H24A—C24—H24B	109.5
O1—C8—C9	116.4 (2)	C23—C24—H24C	109.5
C8—C9—H9A	109.5	H24A—C24—H24C	109.5
C8—C9—H9B	109.5	H24B—C24—H24C	109.5
H9A—C9—H9B	109.5	C30—C25—C26	111.8 (2)
C8—C9—H9C	109.5	C30—C25—S2	113.65 (16)
H9A—C9—H9C	109.5	C26—C25—S2	107.45 (15)
H9B—C9—H9C	109.5	C30—C25—H25	107.9
C11—C10—C15	111.8 (2)	C26—C25—H25	107.9
C11—C10—S1	109.71 (17)	S2—C25—H25	107.9
C15—C10—S1	108.77 (17)	C27—C26—C25	110.0 (2)
C11—C10—H10	108.8	C27—C26—H26A	109.7
C15—C10—H10	108.8	C25—C26—H26A	109.7
S1—C10—H10	108.8	C27—C26—H26B	109.7
C10—C11—C12	110.9 (2)	C25—C26—H26B	109.7
C10—C11—H11A	109.5	H26A—C26—H26B	108.2
C12—C11—H11A	109.5	C28—C27—C26	110.9 (2)
C10—C11—H11B	109.5	C28—C27—H27A	109.4
C12—C11—H11B	109.5	C26—C27—H27A	109.4
H11A—C11—H11B	108.0	C28—C27—H27B	109.4
C13—C12—C11	111.1 (3)	C26—C27—H27B	109.4
C13—C12—H12A	109.4	H27A—C27—H27B	108.0
C11—C12—H12A	109.4	C27—C28—C29	110.9 (2)
C13—C12—H12B	109.4	C27—C28—H28A	109.5
C11—C12—H12B	109.4	C29—C28—H28A	109.5
H12A—C12—H12B	108.0	C27—C28—H28B	109.5
C14—C13—C12	110.5 (2)	C29—C28—H28B	109.5
C14—C13—H13A	109.6	H28A—C28—H28B	108.0
C12—C13—H13A	109.6	C28—C29—C30	111.3 (2)
C14—C13—H13B	109.6	C28—C29—H29A	109.4
C12—C13—H13B	109.6	C30—C29—H29A	109.4
H13A—C13—H13B	108.1	C28—C29—H29B	109.4
C13—C14—C15	111.4 (2)	C30—C29—H29B	109.4
C13—C14—H14A	109.4	H29A—C29—H29B	108.0
C15—C14—H14A	109.4	C25—C30—C29	110.5 (2)
C13—C14—H14B	109.4	C25—C30—H30A	109.6
C15—C14—H14B	109.4	C29—C30—H30A	109.6
H14A—C14—H14B	108.0	C25—C30—H30B	109.6
C14—C15—C10	109.8 (2)	C29—C30—H30B	109.6
C14—C15—H15A	109.7	H30A—C30—H30B	108.1
			
O2—S1—C1—C8	144.0 (2)	O4—S2—C16—C23	−152.5 (2)
C10—S1—C1—C8	−105.3 (2)	C25—S2—C16—C23	95.1 (2)
O2—S1—C1—C2	−35.5 (2)	O4—S2—C16—C17	25.0 (2)
C10—S1—C1—C2	75.1 (2)	C25—S2—C16—C17	−87.4 (2)
C8—C1—C2—C3	177.8 (2)	C23—C16—C17—C18	177.1 (3)
S1—C1—C2—C3	−2.5 (4)	S2—C16—C17—C18	−0.7 (4)
C8—C1—C2—C7	−0.7 (2)	C23—C16—C17—C22	−1.5 (2)
S1—C1—C2—C7	178.90 (17)	S2—C16—C17—C22	−179.34 (18)
C7—C2—C3—C4	−0.2 (3)	C22—C17—C18—C19	−0.5 (3)
C1—C2—C3—C4	−178.6 (2)	C16—C17—C18—C19	−179.0 (2)
C2—C3—C4—C5	−0.5 (3)	C17—C18—C19—C20	−0.9 (4)
C2—C3—C4—Br1	179.47 (16)	C17—C18—C19—Br2	177.68 (16)
C3—C4—C5—C6	0.0 (4)	C18—C19—C20—C21	1.4 (4)
Br1—C4—C5—C6	−179.90 (18)	Br2—C19—C20—C21	−177.15 (19)
C4—C5—C6—C7	1.0 (3)	C19—C20—C21—C22	−0.5 (4)
C8—O1—C7—C6	−179.9 (2)	C23—O3—C22—C21	−179.3 (2)
C8—O1—C7—C2	−0.7 (2)	C23—O3—C22—C17	0.4 (3)
C5—C6—C7—O1	177.4 (2)	C20—C21—C22—O3	178.7 (2)
C5—C6—C7—C2	−1.6 (4)	C20—C21—C22—C17	−0.9 (4)
C3—C2—C7—O1	−177.94 (19)	C18—C17—C22—O3	−178.2 (2)
C1—C2—C7—O1	0.9 (2)	C16—C17—C22—O3	0.7 (2)
C3—C2—C7—C6	1.3 (4)	C18—C17—C22—C21	1.4 (4)
C1—C2—C7—C6	−179.9 (2)	C16—C17—C22—C21	−179.6 (2)
C2—C1—C8—O1	0.3 (3)	C17—C16—C23—O3	1.9 (3)
S1—C1—C8—O1	−179.31 (15)	S2—C16—C23—O3	179.85 (15)
C2—C1—C8—C9	−175.8 (2)	C17—C16—C23—C24	−178.5 (2)
S1—C1—C8—C9	4.5 (4)	S2—C16—C23—C24	−0.5 (4)
C7—O1—C8—C1	0.2 (2)	C22—O3—C23—C16	−1.4 (3)
C7—O1—C8—C9	177.06 (19)	C22—O3—C23—C24	178.9 (2)
O2—S1—C10—C11	179.02 (18)	O4—S2—C25—C30	−48.50 (19)
C1—S1—C10—C11	68.28 (19)	C16—S2—C25—C30	63.80 (19)
O2—S1—C10—C15	−58.37 (19)	O4—S2—C25—C26	75.73 (18)
C1—S1—C10—C15	−169.11 (17)	C16—S2—C25—C26	−171.97 (17)
C15—C10—C11—C12	54.9 (3)	C30—C25—C26—C27	−56.7 (3)
S1—C10—C11—C12	175.7 (2)	S2—C25—C26—C27	177.98 (17)
C10—C11—C12—C13	−54.7 (4)	C25—C26—C27—C28	57.1 (3)
C11—C12—C13—C14	56.2 (4)	C26—C27—C28—C29	−57.3 (3)
C12—C13—C14—C15	−58.1 (3)	C27—C28—C29—C30	56.1 (3)
C13—C14—C15—C10	57.5 (3)	C26—C25—C30—C29	55.7 (3)
C11—C10—C15—C14	−56.0 (3)	S2—C25—C30—C29	177.52 (16)
S1—C10—C15—C14	−177.3 (2)	C28—C29—C30—C25	−55.0 (3)

### Hydrogen-bond geometry (Å, °)

**Table:**

*D*—H···*A*	*D*—H	H···*A*	*D*···*A*	*D*—H···*A*
C5—H5···O2^i^	0.95	2.56	3.495 (3)	170
C25—H25···O2	1.00	2.57	3.409 (3)	142

Symmetry codes: (i) *x*, *y*+1, *z*.
